# Synthesis, crystal structure, DFT calculations and Hirshfeld surface analysis of 3-butyl-2,6-bis­(4-fluoro­phen­yl)piperidin-4-one

**DOI:** 10.1107/S2056989020004636

**Published:** 2020-04-09

**Authors:** K. Anitha, S. Sivakumar, R. Arulraj, K. Rajkumar, Manpreet Kaur, Jerry P. Jasinski

**Affiliations:** a Research and Development Centre, Bharathiar University, Coimbatore, Tamilnadu 641 046, India; b Department of Chemistry, Thiruvalluvar Arts and Science College, Kurinjipadi, Tamilnadu 607 302, India; c Department of Electrical and Computer Engineering, National University of Singapore, Singapore 117 583; d Department of Chemistry, Keene State College, 229 Main Street, Keene, NH 03435-2001, USA

**Keywords:** piperidin-4-one, crystal structure, Hirshfeld surface

## Abstract

The title compound, C_21_H_23_F_2_NO, consists of two fluoro­phenyl groups and one butyl group equatorially oriented on a piperidine ring, which adopts a chair conformation. The dihedral angle between the mean planes of the phenyl rings is 72.1 (1)°. In the crystal, N—H⋯O and weak C—H⋯F inter­actions, which form *R* ^2^_2_[14] motifs, link the mol­ecules into infinite *C*(6) chains propagating along [001]. A weak C—H⋯π inter­action is also observed. A Hirshfeld surface analysis of the crystal structure indicates that the most significant contributions to the crystal packing are from H⋯H (53.3%), H⋯C/C⋯H (19.1%), H⋯F/F⋯H (15.7%) and H⋯O/O⋯H (7.7%) contacts. Density functional theory geometry-optimized calculations were compared to the experimentally determined structure in the solid state and used to determine the HOMO–LUMO energy gap and compare it to the UV–vis experimental spectrum.

## Abstract 2

The title compound consists of two fluoro­phenyl groups and one butyl group equatorially oriented on a piperidine ring, which adopts a chair conformation. The dihedral angle between the mean planes of the phenyl rings is 72.1 (1)°. In the crystal, weak N—H⋯O and C—H⋯F inter­actions, which form 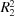
[14] motifs, link the mol­ecules into infinite *C*(6) chains propgagating along [001].

## Chemical context   

Piperidin-4-one compounds have various biological properties and have applications as anti-viral, antitumor, and antihistaminic agents (El-Subbagh *et al.*, 2000 ▸; Mobio *et al.*, 1989 ▸; Katritzky & Fan, 1990 ▸; Arulraj *et al.*, 2020 ▸). 2,6-Disubstituted piperidine-4-ones commonly adopt a chair conformation for the heterocyclic ring (see, for example, Rajkumar *et al.*, 2018 ▸). However, on varying the substituents attached to the phenyl ring, the conformation of the ring may change (*e.g*. Ramachandran *et al.*, 2007 ▸; Arulraj *et al.*, 2020 ▸). Additionally, the attached functional group on the crystalline compound is important to determine the activity of the compound in the area of drug discovery.
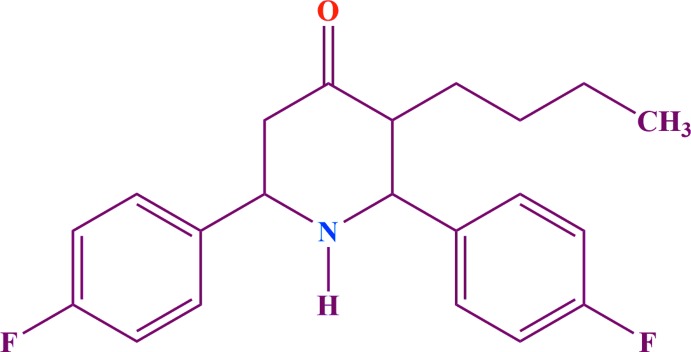


As part of our studies in this area, we now describe the synthesis and structure of the title compound, C_21_H_23_F_2_NO, (I), in order to establish the structural effects of the butyl and fluoro groups on the conformation. DFT calculations and a Hirshfeld analysis have also been carried out.

## Structural commentary   

**Figure 1:**
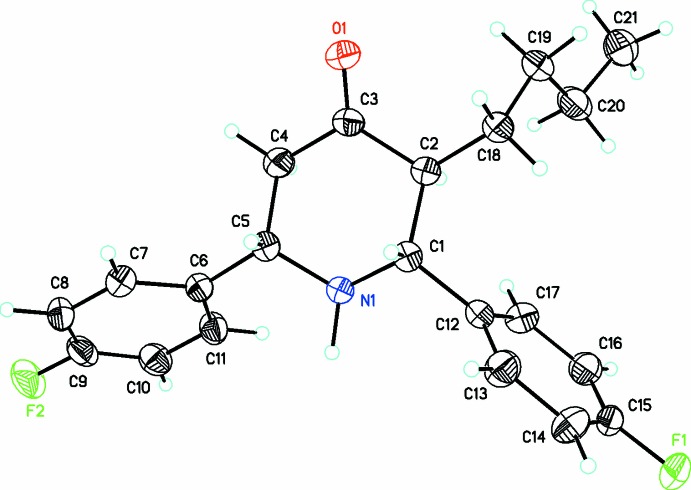
A view of the mol­ecular structure of C_21_H_23_F_2_NO, showing displacement ellipsoids drawn at the 30% probability level.

Compound (I) crystallizes in space group *P2_1_/c* with one mol­ecule in the asymmetric unit (Fig. 1 ▸). In the arbitrarily chosen asymmetric unit, the stereogenic centres have the following configurations: C1 *S*, C2 *R* and C5 *R*, but crystal symmetry generates a racemic mixture. The piperidine ring adopts a slightly distorted chair conformation with puckering parameters *Q* = 0.5864 (16) Å, θ = 6.56 (15)°, φ = 356.9 (14)°. The dihedral angles for the C1–C5/N1 (all atoms) piperidine (*A*), C6–C11 fluorophenyl (*B*) and C12–C17 fluorophenyl (*C*) rings are *A*/*B* = 65.50 (8), *A*/*C* = 73.87 (8) and *B*/*C* = 72.11 (8)°. The substituents on the piperidine ring adopt equatorial orientations with the keto oxygen atom being anti-clinal [O1—C3—C2—C1 = −124.44 (16)°]. The butyl group lies in a syn-periplanar orientation [O1—C3—C2—C18 = 0.7 (2)] while the fluoro­phenyl groups are both anti-clinal [N1—C5—C6—C7 = −148.28 (13) and N1—C1—C12—C17 = −75.42 (16)°]. The sum of the bond angles around N1 is 336.8°, which is consistent with *sp* ^3^ hybridization for this atom (Beddoes *et al.*, 1986 ▸).

## Supra­molecular features   

**Figure 2:**
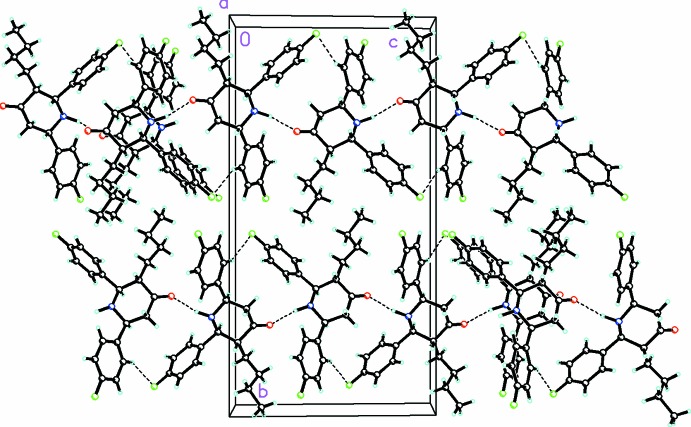
Crystal packing for C_21_H_23_F_2_NO viewed along the *a*-axis direction. Dashed lines indicate N—H⋯O hydrogen bonds and weak C—H⋯F inter­actions forming 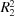
(14) loops and infinite **C**(6) chains (*via* the N—H⋯O bond) along the *c-*axis direction.

N1—H1⋯O1 and weak C7—H7⋯F1 inter­actions are observed in the crystal of (I) (Table 1 ▸, Fig. 2 ▸), which form 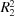
[14] graph-set ring motifs and infinite *C*(6) chains (*via* the N—H⋯O bond) along [001]. Some longer C—H⋯O and C—H⋯F contacts are also present as well as a single weak C—H⋯π inter­action (Table 1 ▸).

## Hirshfeld surface analysis   

**Figure 3:**
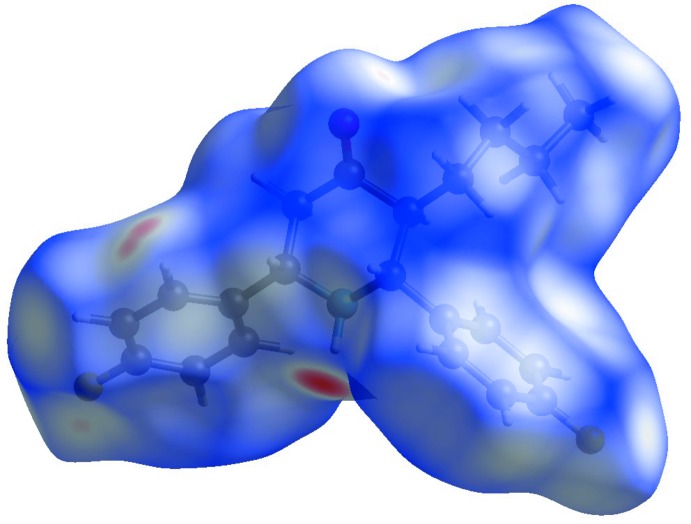
A view of the three-dimensional Hirshfeld surface for C_21_H_23_F_2_NO, plotted over *d*_norm_ in the range −0.39 to 1.31 a.u.

A Hirshfeld surface (HS) analysis (Spackman & Jayatilaka, 2009 ▸) was carried out using *CrystalExplorer17.5* (Turner *et al.*, 2017 ▸) to visualize the inter­molecular inter­actions in (I). The bright-red spot near H1 indicates its role as a hydrogen-bond donor to O1 (Fig. 3 ▸) and another red region near H7 correlates with the C7—H7⋯F1 inter­action. The shape-index of the HS represents a way to visualize π–π stacking by the presence of red and/or blue triangles but there are none in in the title compound (see Figure S1 in the supporting information). The curvedness of the HS can be used to divide the mol­ecular surface into contact patches with each neighbouring mol­ecule thereby using it to define a coordination number in the crystal (see Figure S2 in the supporting information).

**Figure 4:**
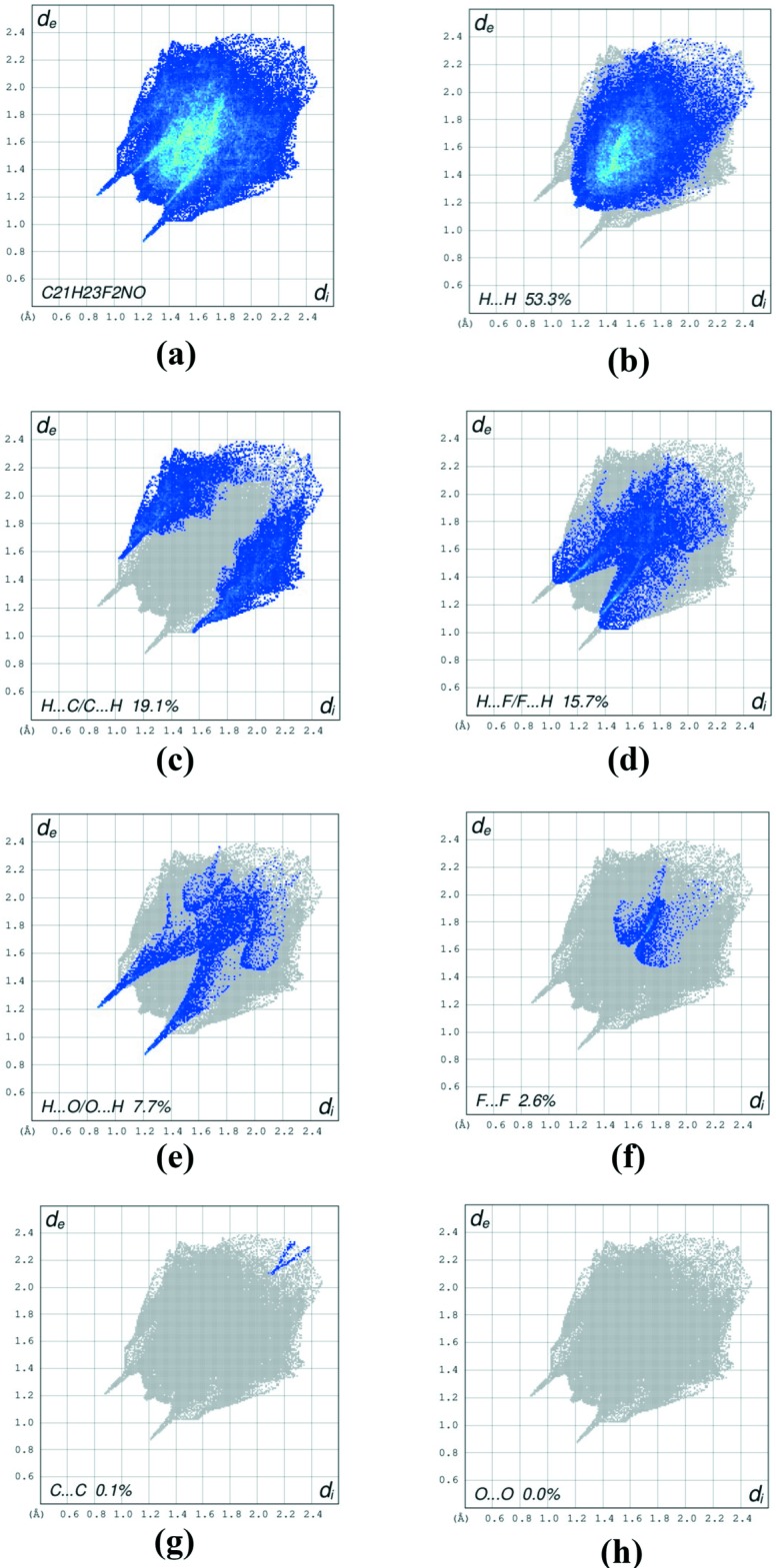
A view of the two-dimensional fingerprint plots for C_21_H_23_F_2_NO, showing (*a*) all inter­actions, and separated into (*b*) H⋯H, (*c*) H⋯C/C⋯H, (*d*) H⋯F/F⋯H, (*e*) O⋯H/H⋯O, (*f*) F⋯F, (*g*) C—C and (*h*) O⋯O inter­actions. The *d*_i_ and *d*_e_ values are the closest inter­nal and external distances (in Å) from given points on the Hirshfeld surface contacts.

Two-dimensional fingerprint plots show the relative contributions of the various types of contacts to the Hirshfeld surface for (I) (McKinnon *et al.*, 2007 ▸). The overal plot is shown in Fig. 4 ▸ *a*. The H⋯H contacts (53.3%) are the most important inter­actions (Fig. 4 ▸ *b*), presumably because of the large hydrogen content of (I), with a pair of blue-coloured blunt spikes directing towards the bottom left, in the region 1.20 Å < (*d*_e_ + *d*_i_) < 1.19 Å. The pair of wings for the H⋯C/C⋯H contacts (Fig. 4 ▸ *c*; 19.1% contribution to the HS) is in the region 1.04 Å < (*d*_e_ + *d*_i_) < 1.58 Å and includes the weak C—H⋯π inter­action. The H⋯F/F⋯H contacts (Fig. 4 ▸ *d*; 15.7% contribution) are seen as a pair of wings in the region 1.04 Å < (*d*_e_ + *d*_i_) < 1.38 Å. The wings for the H⋯O/O⋯H contacts (Fig. 4 ▸ *e*; 7.7% contribution) are in the region of 0.88 Å < (*d*_e_ + *d*_i_) < 1.20 Å while the blunt wings in the plot for F⋯F contacts (Fig. 4 ▸ *f*; 2.6%) are in the region 1.60 Å < (*d*_e_ + *d*_i_) < 1.70 Å. The C⋯C contacts (Fig. 4 ▸ *g*) make a negligible 0.1% contribution and are viewed as a dash pattern pointing diagonally left. The O⋯O contacts (Fig. 4 ▸ *h*) make no contribution to the HS. The most significant of these contributions to the overall Hirshfeld surface are shown in Figure S3 in the supporting information.

## DFT Calculations   

A density functional theory (DFT) geometry-optimized calculation for (I) was carried out using *WebMo Pro* (Schmidt & Polik, 2007 ▸) in the *GAUSSIAN 09* program package (Frisch *et al.*, 2009 ▸) using the 6-31+G(d) basis set (Hehre *et al.*, 1986 ▸). The starting geometry was taken from the crystal structure and no solvent correction was applied. A comparison of bond angles and bond distances in the crystal to those from the DFT calculation are listed in supplementary Table S1, which generally shows good agreement. An overlay of the geometry-optimized calculation with the crystal structure has an r.m.s. deviation of 0.478 Å. The major difference between the experimental and calculated structures occurs in the orientation of the C12–C17 rings, which are rotated by 41.8 (6)° with respect to each other.

**Figure 5:**
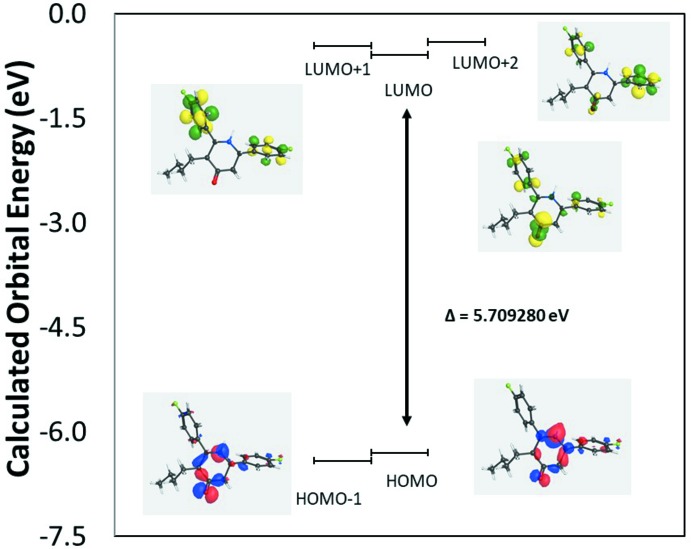
Schematic MO diagram.

**Figure 6:**
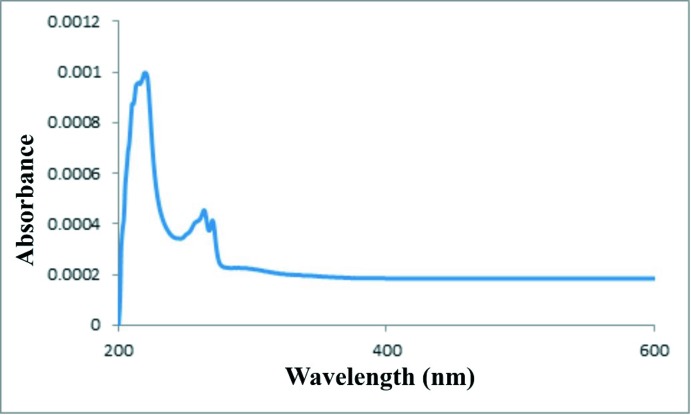
UV–vis spectrum of C_21_H_23_F_2_NO

The calculated energies (eV) for the frontier mol­ecular orbitals are shown in Fig. 5 ▸ and key parameters are listed in supplementary Table S2. Both the HOMO and HOMO−1 are localized largely on the piperidine ring. For the LUMO, LUMO+1 and LUMO+2, the orbitals are delocalized over the piperidine ring as well as both phenyl rings. The observed UV/vis absorption spectrum (Fig. 6 ▸) shows two band envelopes with λ_max_ values located at *ca* 256 and 216 nm (∼4.84 and 5.74 eV). The molar extinction coefficients, ∊, are 1.12 and 2.50 l mol^−1^ cm^−1^, respectively. We tentatively assign the first absorption band envelope at 256 nm to overlapping contributions from HOMO → LUMO (energy gap 5.71 eV), HOMO → LUMO+1 (5.83 eV) and HOMO−1 → LUMO (5.82 eV). The band at 216 nm is assigned to overlapping contributions from HOMO → LUMO+2 (5.89 eV), HOMO−1 → LUMO+1 (5.95 eV) and HOMO−1 → LUMO+2 (6.01 eV).

## Database survey   

A search in the Cambridge Crystallographic Database (CSD version 2.0.4 of December 2019; Groom *et al.*. 2016 ▸) for the 2,6-di­phenyl­piperidin-4-one skeleton resulted in 240 hits, which was refined to 44 matches by removing those structures in which the title skeleton substructure was combined with larger mol­ecules. The four most closely related remaining structures based on the pendant arms of the 2,6 di­phenyl­piperidine-4-one central substructure are 2,6-diphenyl-3-iso­propyl­piperidin-4-one (ACEZUD; Nilofar Nissa *et al.*, 2001 ▸), *t*(3)-pentyl-*r*(2),*c*6)-di­phenyl­piperidin-4-one (RUGLOV; Gayathri *et al.*, 2009 ▸), 3-(2-chloro­eth­yl)-*r*(2),*c*(6)-di­phenyl­piperidin-4-one (PEXDII; Rajkumar *et al.*, 2018 ▸) and 3-(2-chloro­eth­yl)-*r*(2),*c*(6)-bis­(4-fluoro­phen­yl)piperidin-4-one (PEXDOO; Rajkumar *et al.*, 2018 ▸). The piperidine ring in the title compound is in a slightly distorted chair conformation, similar to that observed in ACEZUD and PEXDOO but different from the chair conformation seen in RUGLOV and PEXDII. The dihedral angle between the mean planes of pendant phenyl rings is 72.(1)° in the title compound compared to 76.1 (1)° in PEXDOO, whereas it is 59.90 (5), 59.1 (1) and 63.4 (1)° in RUGLOV, PEXDII and ACEZUD, respectively. In all five compounds, various N—H⋯O and weak C—H⋯O, C—H⋯π or C—H⋯F inter­actions occur in the crystal.

## Synthesis and crystallization   

A mixture of ammonium acetate (0.100 mol, 7.71 g), 4-fluoro­benzaldehyde (0.200 mol, 22.0 ml) and 2-hepta­none (0.100 mol, 14.2 ml) in distilled ethanol was heated first to boiling. After cooling, the viscous liquid obtained was dissolved in ether (200 ml) and shaken with 100 ml concentrated hydro­chloric acid. The precipitated hydro­chloride of 3-butyl-2,6-bis­(4-fluoro­phen­yl)piperidin-4-one was removed by filtration and washed first with a 50 ml mixture of ethanol and ether (1:1) and then with ether to remove most of the coloured impurities. The resulting yellowish base was liberated from an alcoholic solution by adding aqueous ammonia (15 ml) and then diluted with water (200 ml). Then, 1.0 g of the crude sample was dissolved in 100 ml of absolute alcohol, warmed until the sample dissolved, and 2.0 g of animal charcoal added in the resulting solution. The hot solution was filtered and the procedure repeated again. The filtered solution was left for 48 h and colourless prisms of (I) were collected in 75% yield. Analysis for C_21_H_23_F_2_NO (%): found C 74.24, H 6.16, N 4.03; calculated C 73.45, H 6.75, N 4.08; melting point 381.5 K.
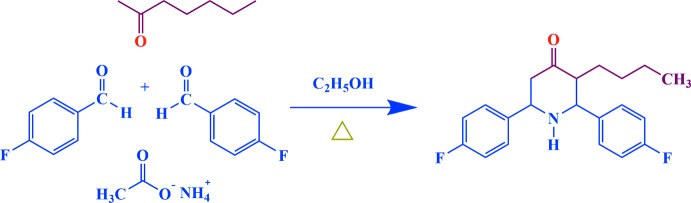


FT–IR (cm^−1^) (KBr): 3287 (ν_N—H_), 3134, 2929, 2866 (ν_C—H_), 1702 (ν_C=O_), 1605, 1508 (ν_C=C_), 793 (ν_C—Cl_); ^1^H NMR (400 MHz, CDCl_3_): δ 7.01–7.45 (*m*, aromatic protons), 4.04 (*d*, H6 proton), 3.68 (*s*, H2 proton), 2.67 (*t*, H5*a* proton), 2.56 (*dd*, H5*e* proton), 2.0 (NH proton), 0.95–1.0 CH_2_(3), 1.09–1.15 CH_2_(2), 1.59–1.63 CH_2_(1), 0.74, (*t*, CH_3_ alkyl proton); ^13^C NMR (400 MHz, CDCl_3_): δ 129.16, 129.38, 128.18, 128.10, 115.64, 115.56, 115.43, 115.35 (aromatic carbon atoms), 138.52 and 137.64 (aromatic *ipso* carbon atoms), 66.33 (C2), 57.50 (C3), 208.7 (C4), 51.63 (C5), 61.08 (C6), 24.30 C18H_2_, 29.71 C19H_2_, 22.75 C20H_2_, 13.81 C21H_3_.

## Refinement   

Crystal data, data collection and structure refinement details are summarized in Table 2 ▸. The C-bound H atoms were geometrically placed (C—H = 0.93–0.98 Å) and refined as riding atoms. The N-bound H atom was located in a difference map and its position was fixed. The methyl group was allowed to rotate, but not to tip, to best fit the electron density. The constraint *U*_iso_(H) = 1.2*U*_eq_(carrier) or 1.5*U*_eq_(methyl carrier) was applied in all cases.

## Supplementary Material

Crystal structure: contains datablock(s) global, I. DOI: 10.1107/S2056989020004636/hb7882sup1.cif

Structure factors: contains datablock(s) I. DOI: 10.1107/S2056989020004636/hb7882Isup2.hkl

Theoretical chemistry data and Hirshfeld figures. DOI: 10.1107/S2056989020004636/hb7882sup3.docx

CCDC reference: 1994539

Additional supporting information:  crystallographic information; 3D view; checkCIF report

## Figures and Tables

**Table 1:** Hydrogen-bond geometry (Å, °) *Cg*3 is the centroid of the C12–C17 ring.

*D*—H⋯*A*	*D*—H	H⋯*A*	*D*⋯*A*	*D*—H⋯*A*
N1—H1⋯O1^i^	1.05	2.06	3.0921 (16)	165
C7—H7⋯F1^ii^	0.95	2.52	3.3291 (18)	143
C10—H10⋯O1^iii^	0.95	2.66	3.470 (2)	144
C16—H16⋯F2^iv^	0.95	2.62	3.3680 (18)	136
C21—H21*C*⋯F2^ii^	0.98	2.58	3.489 (2)	154
C21—H21*A*⋯*Cg*3^v^	0.98	2.95	3.793 (2)	145

Symmetry codes: (i) 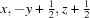; (ii) 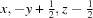; (iii) 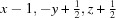; (iv) 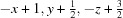; (v) 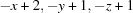.

**Table 2:** Experimental details

Crystal data	
Chemical formula	C_21_H_23_F_2_NO
*M*_r_	343.40
Crystal system, space group	Monoclinic, *P*2_1_/*c*
Temperature (K)	173
*a*, *b*, *c* (Å)	5.4945 (3), 25.0707 (13), 12.9811 (9)
β (°)	93.497 (6)
*V* (Å^3^)	1784.83 (18)
*Z*	4
Radiation type	Cu *K*α
μ (mm^−1^)	0.76
Crystal size (mm)	0.42 × 0.36 × 0.35

Data collection	
Diffractometer	Rigaku Oxford Diffraction Gemini Eos
Absorption correction	Multi-scan (*CrysAlis PRO*; Rigaku OD, 2019 ▸)
*T*_min_, *T*_max_	0.803, 1.000
No. of measured, independent and observed [*I* > 2σ(*I*)] reflections	6900, 3404, 3045
*R*_int_	0.027
(sin θ/λ)_max_ (Å^−1^)	0.614

Refinement	
*R*[*F* ^2^ > 2σ(*F* ^2^)], *wR*(*F* ^2^), *S*	0.045, 0.126, 1.04
No. of reflections	3404
No. of parameters	228
H-atom treatment	H-atom parameters constrained
Δρ_max_, Δρ_min_ (e Å^−3^)	0.26, −0.24

Computer programs: *CrysAlis PRO* (Rigaku OD, 2019 ▸), *SHELXT* (Sheldrick, 2015*a* ▸), *SHELXL* (Sheldrick, 2015*b* ▸) and *OLEX2* (Dolomanov *et al.*, 2009 ▸).

## supplementary crystallographic information

### Crystal data

**Table:**

C_21_H_23_F_2_NO	*F*(000) = 728
*M**_r_* = 343.40	*D*_x_ = 1.278 Mg m^−^^3^
Monoclinic, *P*2_1_/*c*	Cu *K*α radiation, λ = 1.54184 Å
*a* = 5.4945 (3) Å	Cell parameters from 3131 reflections
*b* = 25.0707 (13) Å	θ = 0.8–1.0°
*c* = 12.9811 (9) Å	µ = 0.76 mm^−^^1^
β = 93.497 (6)°	*T* = 173 K
*V* = 1784.83 (18) Å^3^	Prism, colourless
*Z* = 4	0.42 × 0.36 × 0.35 mm

### Data collection

**Table:**

Rigaku Oxford Diffraction Gemini Eos diffractometer	3404 independent reflections
Radiation source: fine-focus sealed X-ray tube	3045 reflections with *I* > 2σ(*I*)
Detector resolution: 16.0416 pixels mm^-1^	*R*_int_ = 0.027
ω scans	θ_max_ = 71.3°, θ_min_ = 3.5°
Absorption correction: multi-scan (CrysAlisPro; Rigaku OD, 2019)	*h* = −6→6
*T*_min_ = 0.803, *T*_max_ = 1.000	*k* = −30→26
6900 measured reflections	*l* = −9→15

### Refinement

**Table:**

Refinement on *F*^2^	Hydrogen site location: mixed
Least-squares matrix: full	H-atom parameters constrained
*R*[*F*^2^ > 2σ(*F*^2^)] = 0.045	*w* = 1/[σ^2^(*F*_o_^2^) + (0.0664*P*)^2^ + 0.4364*P*] where *P* = (*F*_o_^2^ + 2*F*_c_^2^)/3
*wR*(*F*^2^) = 0.126	(Δ/σ)_max_ < 0.001
*S* = 1.04	Δρ_max_ = 0.26 e Å^−^^3^
3404 reflections	Δρ_min_ = −0.24 e Å^−^^3^
228 parameters	Extinction correction: SHELXL (Sheldrick 2015b), Fc^*^=kFc[1+0.001xFc^2^λ^3^/sin(2θ)]^-1/4^
0 restraints	Extinction coefficient: 0.0035 (5)
Primary atom site location: dual	

### Special details

**Table:**

Geometry. All esds (except the esd in the dihedral angle between two l.s. planes) are estimated using the full covariance matrix. The cell esds are taken into account individually in the estimation of esds in distances, angles and torsion angles; correlations between esds in cell parameters are only used when they are defined by crystal symmetry. An approximate (isotropic) treatment of cell esds is used for estimating esds involving l.s. planes.

### Fractional atomic coordinates and isotropic or equivalent isotropic displacement parameters (Å^2^)

**Table:**

	*x*	*y*	*z*	*U*_iso_*/*U*_eq_	
F1	1.1088 (2)	0.45264 (4)	0.91794 (7)	0.0458 (3)	
F2	0.2626 (2)	0.04577 (4)	0.65950 (9)	0.0493 (3)	
O1	0.9789 (3)	0.28871 (5)	0.32525 (9)	0.0439 (3)	
N1	0.8301 (2)	0.26447 (5)	0.61588 (9)	0.0265 (3)	
H1	0.859899	0.250816	0.692480	0.032*	
C1	1.0000 (3)	0.30675 (6)	0.58925 (11)	0.0267 (3)	
H1A	1.163280	0.290264	0.580635	0.032*	
C2	0.9099 (3)	0.33248 (6)	0.48525 (11)	0.0278 (3)	
H2	0.745757	0.348399	0.494336	0.033*	
C3	0.8784 (3)	0.28808 (6)	0.40558 (11)	0.0312 (3)	
C4	0.7247 (3)	0.24148 (6)	0.43663 (12)	0.0341 (4)	
H4A	0.729221	0.212850	0.384262	0.041*	
H4B	0.553231	0.252968	0.440962	0.041*	
C5	0.8253 (3)	0.22036 (6)	0.54247 (11)	0.0282 (3)	
H5	0.996468	0.207883	0.535604	0.034*	
C6	0.6767 (3)	0.17398 (6)	0.57869 (11)	0.0261 (3)	
C7	0.7422 (3)	0.12243 (6)	0.55255 (12)	0.0303 (3)	
H7	0.883737	0.116949	0.515379	0.036*	
C8	0.6046 (3)	0.07877 (6)	0.57975 (13)	0.0344 (4)	
H8	0.649053	0.043603	0.561167	0.041*	
C9	0.4023 (3)	0.08793 (6)	0.63432 (12)	0.0340 (4)	
C10	0.3355 (3)	0.13790 (7)	0.66510 (13)	0.0351 (4)	
H10	0.198118	0.142774	0.705110	0.042*	
C11	0.4740 (3)	0.18125 (6)	0.63626 (12)	0.0314 (3)	
H11	0.429534	0.216223	0.656111	0.038*	
C12	1.0247 (3)	0.34625 (6)	0.67737 (11)	0.0265 (3)	
C13	1.2301 (3)	0.34492 (6)	0.74451 (13)	0.0350 (4)	
H13	1.353733	0.319324	0.734112	0.042*	
C14	1.2594 (3)	0.38022 (7)	0.82671 (13)	0.0389 (4)	
H14	1.400409	0.378901	0.872784	0.047*	
C15	1.0794 (3)	0.41700 (6)	0.83961 (12)	0.0327 (4)	
C16	0.8701 (3)	0.41935 (6)	0.77634 (12)	0.0347 (4)	
H16	0.747117	0.444956	0.787708	0.042*	
C17	0.8432 (3)	0.38332 (6)	0.69539 (12)	0.0312 (3)	
H17	0.698502	0.383920	0.651444	0.037*	
C18	1.0774 (3)	0.37699 (6)	0.45103 (12)	0.0300 (3)	
H18A	1.214626	0.360763	0.416194	0.036*	
H18B	1.146589	0.395904	0.513051	0.036*	
C19	0.9510 (3)	0.41772 (6)	0.37807 (12)	0.0347 (4)	
H19A	1.075666	0.442006	0.352266	0.042*	
H19B	0.873544	0.398610	0.317901	0.042*	
C20	0.7587 (3)	0.45074 (7)	0.42774 (14)	0.0409 (4)	
H20A	0.828276	0.464904	0.494378	0.049*	
H20B	0.619586	0.427418	0.442398	0.049*	
C21	0.6662 (4)	0.49699 (7)	0.36054 (15)	0.0453 (4)	
H21A	0.800002	0.522000	0.351000	0.068*	
H21B	0.534931	0.515376	0.394092	0.068*	
H21C	0.603886	0.483414	0.293211	0.068*	

### Atomic displacement parameters (Å^2^)

**Table:**

	*U*^11^	*U*^22^	*U*^33^	*U*^12^	*U*^13^	*U*^23^
F1	0.0703 (7)	0.0351 (5)	0.0321 (5)	−0.0142 (5)	0.0041 (5)	−0.0113 (4)
F2	0.0580 (6)	0.0342 (5)	0.0559 (7)	−0.0176 (5)	0.0041 (5)	0.0125 (5)
O1	0.0700 (8)	0.0380 (6)	0.0248 (6)	−0.0140 (6)	0.0122 (5)	−0.0041 (5)
N1	0.0356 (6)	0.0226 (6)	0.0212 (6)	−0.0038 (5)	0.0004 (5)	0.0010 (4)
C1	0.0301 (7)	0.0244 (7)	0.0255 (7)	−0.0014 (5)	0.0015 (5)	0.0005 (6)
C2	0.0342 (7)	0.0259 (7)	0.0233 (7)	−0.0032 (6)	0.0018 (6)	0.0009 (6)
C3	0.0411 (8)	0.0301 (7)	0.0219 (7)	−0.0048 (6)	−0.0009 (6)	0.0028 (6)
C4	0.0449 (9)	0.0313 (8)	0.0258 (8)	−0.0093 (6)	−0.0010 (6)	−0.0008 (6)
C5	0.0340 (7)	0.0244 (7)	0.0262 (7)	−0.0035 (6)	0.0026 (6)	0.0002 (6)
C6	0.0309 (7)	0.0227 (7)	0.0244 (7)	−0.0013 (5)	−0.0010 (5)	0.0010 (5)
C7	0.0353 (7)	0.0276 (7)	0.0281 (7)	0.0002 (6)	0.0027 (6)	−0.0029 (6)
C8	0.0467 (9)	0.0216 (7)	0.0344 (8)	0.0011 (6)	−0.0031 (7)	−0.0008 (6)
C9	0.0391 (8)	0.0284 (8)	0.0338 (8)	−0.0087 (6)	−0.0040 (6)	0.0093 (6)
C10	0.0335 (7)	0.0358 (8)	0.0365 (9)	−0.0006 (6)	0.0063 (6)	0.0047 (6)
C11	0.0343 (7)	0.0243 (7)	0.0356 (8)	0.0029 (6)	0.0033 (6)	0.0009 (6)
C12	0.0335 (7)	0.0229 (7)	0.0231 (7)	−0.0047 (5)	0.0029 (5)	0.0025 (5)
C13	0.0371 (8)	0.0334 (8)	0.0338 (8)	0.0041 (6)	−0.0029 (6)	−0.0032 (6)
C14	0.0398 (8)	0.0430 (9)	0.0327 (9)	−0.0049 (7)	−0.0075 (7)	−0.0048 (7)
C15	0.0496 (9)	0.0256 (7)	0.0235 (7)	−0.0119 (6)	0.0064 (6)	−0.0038 (6)
C16	0.0430 (8)	0.0301 (8)	0.0316 (8)	0.0033 (6)	0.0074 (6)	−0.0015 (6)
C17	0.0321 (7)	0.0344 (8)	0.0270 (7)	0.0004 (6)	0.0006 (6)	−0.0012 (6)
C18	0.0348 (7)	0.0290 (7)	0.0266 (7)	−0.0063 (6)	0.0045 (6)	0.0004 (6)
C19	0.0460 (9)	0.0294 (8)	0.0293 (8)	−0.0052 (6)	0.0073 (6)	0.0038 (6)
C20	0.0513 (10)	0.0329 (8)	0.0396 (9)	−0.0004 (7)	0.0114 (8)	0.0073 (7)
C21	0.0581 (11)	0.0334 (9)	0.0442 (10)	0.0035 (8)	0.0022 (8)	0.0044 (7)

### Geometric parameters (Å, º)

**Table:**

F1—C15	1.3559 (17)	C10—H10	0.9500
F2—C9	1.3581 (17)	C10—C11	1.391 (2)
O1—C3	1.2096 (19)	C11—H11	0.9500
N1—H1	1.0549	C12—C13	1.384 (2)
N1—C1	1.4678 (17)	C12—C17	1.393 (2)
N1—C5	1.4590 (18)	C13—H13	0.9500
C1—H1A	1.0000	C13—C14	1.388 (2)
C1—C2	1.5498 (19)	C14—H14	0.9500
C1—C12	1.5128 (19)	C14—C15	1.370 (2)
C2—H2	1.0000	C15—C16	1.373 (2)
C2—C3	1.522 (2)	C16—H16	0.9500
C2—C18	1.5296 (19)	C16—C17	1.387 (2)
C3—C4	1.511 (2)	C17—H17	0.9500
C4—H4A	0.9900	C18—H18A	0.9900
C4—H4B	0.9900	C18—H18B	0.9900
C4—C5	1.543 (2)	C18—C19	1.530 (2)
C5—H5	1.0000	C19—H19A	0.9900
C5—C6	1.5121 (19)	C19—H19B	0.9900
C6—C7	1.389 (2)	C19—C20	1.517 (2)
C6—C11	1.391 (2)	C20—H20A	0.9900
C7—H7	0.9500	C20—H20B	0.9900
C7—C8	1.388 (2)	C20—C21	1.520 (2)
C8—H8	0.9500	C21—H21A	0.9800
C8—C9	1.373 (2)	C21—H21B	0.9800
C9—C10	1.372 (2)	C21—H21C	0.9800
			
C1—N1—H1	113.1	C6—C11—C10	120.76 (14)
C5—N1—H1	111.4	C6—C11—H11	119.6
C5—N1—C1	112.32 (11)	C10—C11—H11	119.6
N1—C1—H1A	108.4	C13—C12—C1	119.50 (13)
N1—C1—C2	109.37 (11)	C13—C12—C17	118.36 (14)
N1—C1—C12	108.87 (11)	C17—C12—C1	122.12 (13)
C2—C1—H1A	108.4	C12—C13—H13	119.3
C12—C1—H1A	108.4	C12—C13—C14	121.38 (15)
C12—C1—C2	113.29 (11)	C14—C13—H13	119.3
C1—C2—H2	107.8	C13—C14—H14	120.9
C3—C2—C1	107.73 (11)	C15—C14—C13	118.19 (15)
C3—C2—H2	107.8	C15—C14—H14	120.9
C3—C2—C18	112.44 (12)	F1—C15—C14	118.78 (15)
C18—C2—C1	113.01 (12)	F1—C15—C16	118.50 (15)
C18—C2—H2	107.8	C14—C15—C16	122.72 (14)
O1—C3—C2	122.51 (14)	C15—C16—H16	120.9
O1—C3—C4	122.15 (14)	C15—C16—C17	118.15 (15)
C4—C3—C2	115.25 (12)	C17—C16—H16	120.9
C3—C4—H4A	109.9	C12—C17—H17	119.4
C3—C4—H4B	109.9	C16—C17—C12	121.17 (14)
C3—C4—C5	109.09 (12)	C16—C17—H17	119.4
H4A—C4—H4B	108.3	C2—C18—H18A	108.7
C5—C4—H4A	109.9	C2—C18—H18B	108.7
C5—C4—H4B	109.9	C2—C18—C19	114.09 (12)
N1—C5—C4	108.21 (12)	H18A—C18—H18B	107.6
N1—C5—H5	108.4	C19—C18—H18A	108.7
N1—C5—C6	111.60 (12)	C19—C18—H18B	108.7
C4—C5—H5	108.4	C18—C19—H19A	108.8
C6—C5—C4	111.70 (12)	C18—C19—H19B	108.8
C6—C5—H5	108.4	H19A—C19—H19B	107.7
C7—C6—C5	119.12 (13)	C20—C19—C18	113.74 (13)
C7—C6—C11	118.74 (14)	C20—C19—H19A	108.8
C11—C6—C5	122.13 (13)	C20—C19—H19B	108.8
C6—C7—H7	119.4	C19—C20—H20A	109.0
C8—C7—C6	121.23 (14)	C19—C20—H20B	109.0
C8—C7—H7	119.4	C19—C20—C21	112.94 (14)
C7—C8—H8	121.0	H20A—C20—H20B	107.8
C9—C8—C7	118.03 (14)	C21—C20—H20A	109.0
C9—C8—H8	121.0	C21—C20—H20B	109.0
F2—C9—C8	118.78 (14)	C20—C21—H21A	109.5
F2—C9—C10	118.38 (15)	C20—C21—H21B	109.5
C10—C9—C8	122.84 (14)	C20—C21—H21C	109.5
C9—C10—H10	120.8	H21A—C21—H21B	109.5
C9—C10—C11	118.34 (15)	H21A—C21—H21C	109.5
C11—C10—H10	120.8	H21B—C21—H21C	109.5
			
F1—C15—C16—C17	178.97 (13)	C4—C5—C6—C11	−88.66 (17)
F2—C9—C10—C11	−177.84 (14)	C5—N1—C1—C2	64.80 (15)
O1—C3—C4—C5	123.39 (17)	C5—N1—C1—C12	−170.93 (11)
N1—C1—C2—C3	−54.89 (15)	C5—C6—C7—C8	−176.87 (13)
N1—C1—C2—C18	−179.73 (12)	C5—C6—C11—C10	177.54 (14)
N1—C1—C12—C13	102.72 (15)	C6—C7—C8—C9	−0.6 (2)
N1—C1—C12—C17	−75.42 (16)	C7—C6—C11—C10	−1.5 (2)
N1—C5—C6—C7	−148.28 (13)	C7—C8—C9—F2	178.51 (13)
N1—C5—C6—C11	32.63 (19)	C7—C8—C9—C10	−1.8 (2)
C1—N1—C5—C4	−64.54 (15)	C8—C9—C10—C11	2.5 (2)
C1—N1—C5—C6	172.17 (11)	C9—C10—C11—C6	−0.7 (2)
C1—C2—C3—O1	−124.44 (16)	C11—C6—C7—C8	2.2 (2)
C1—C2—C3—C4	52.03 (17)	C12—C1—C2—C3	−176.53 (12)
C1—C2—C18—C19	−155.74 (13)	C12—C1—C2—C18	58.63 (16)
C1—C12—C13—C14	−179.62 (14)	C12—C13—C14—C15	−0.5 (3)
C1—C12—C17—C16	−179.67 (14)	C13—C12—C17—C16	2.2 (2)
C2—C1—C12—C13	−135.36 (14)	C13—C14—C15—F1	−178.24 (14)
C2—C1—C12—C17	46.50 (18)	C13—C14—C15—C16	1.7 (2)
C2—C3—C4—C5	−53.09 (18)	C14—C15—C16—C17	−1.0 (2)
C2—C18—C19—C20	66.24 (18)	C15—C16—C17—C12	−1.0 (2)
C3—C2—C18—C19	82.03 (16)	C17—C12—C13—C14	−1.4 (2)
C3—C4—C5—N1	55.98 (16)	C18—C2—C3—O1	0.7 (2)
C3—C4—C5—C6	179.22 (13)	C18—C2—C3—C4	177.20 (13)
C4—C5—C6—C7	90.43 (16)	C18—C19—C20—C21	170.21 (14)

### Hydrogen-bond geometry (Å, º)

*Cg*3 is the centroid of the C12–C17 ring.

**Table:**

*D*—H···*A*	*D*—H	H···*A*	*D*···*A*	*D*—H···*A*
N1—H1···O1^i^	1.05	2.06	3.0921 (16)	165
C7—H7···F1^ii^	0.95	2.52	3.3291 (18)	143
C10—H10···O1^iii^	0.95	2.66	3.470 (2)	144
C16—H16···F2^iv^	0.95	2.62	3.3680 (18)	136
C21—H21*C*···F2^ii^	0.98	2.58	3.489 (2)	154
C21—H21*A*···*Cg*3^v^	0.98	2.95	3.793 (2)	145

Symmetry codes: (i) *x*, −*y*+1/2, *z*+1/2; (ii) *x*, −*y*+1/2, *z*−1/2; (iii) *x*−1, −*y*+1/2, *z*+1/2; (iv) −*x*+1, *y*+1/2, −*z*+3/2; (v) −*x*+2, −*y*+1, −*z*+1.
