# Supplementary material for: Synthesis, crystal structure, DFT calculations and Hirshfeld surface analysis of 3-butyl-2,6-bis­(4-fluoro­phen­yl)piperidin-4-one
Source: Acta Crystallogr E Crystallogr Commun. 2020 Apr 9;76(Pt 5):651–5. doi: 10.1107/S2056989020004636 (PMC7199252; doi:10.1107/S2056989020004636)
Supplement: Supplementary file 3 [file e-76-00651-sup3.docx]

**Table S1**


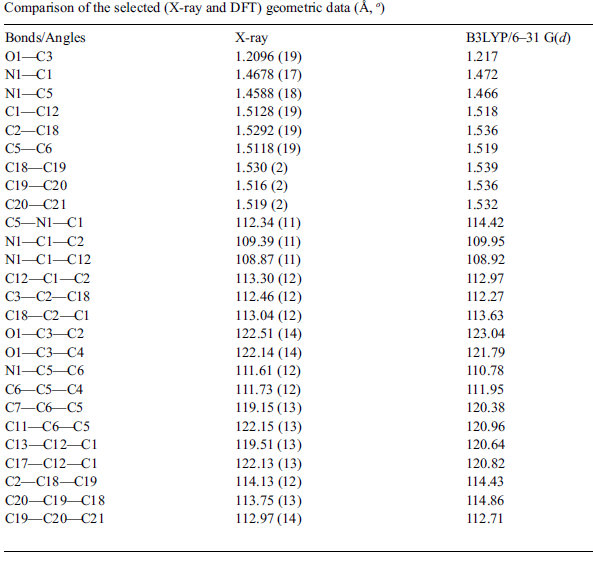


**Table S2: key DFT parameters for (I)**


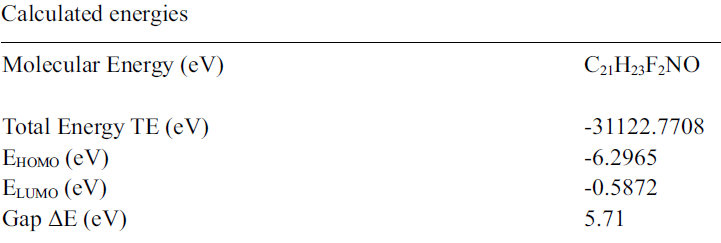

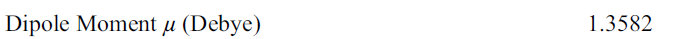


**
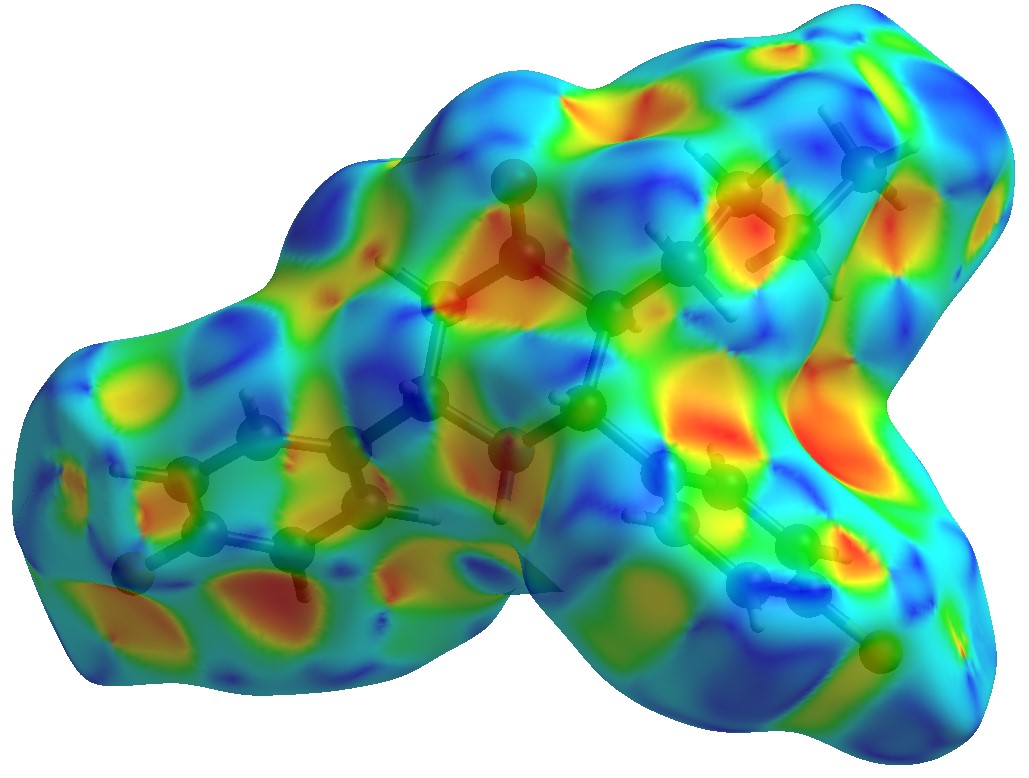
**

**Figure S1: Hirshfeld surface curvature for (I)**

**
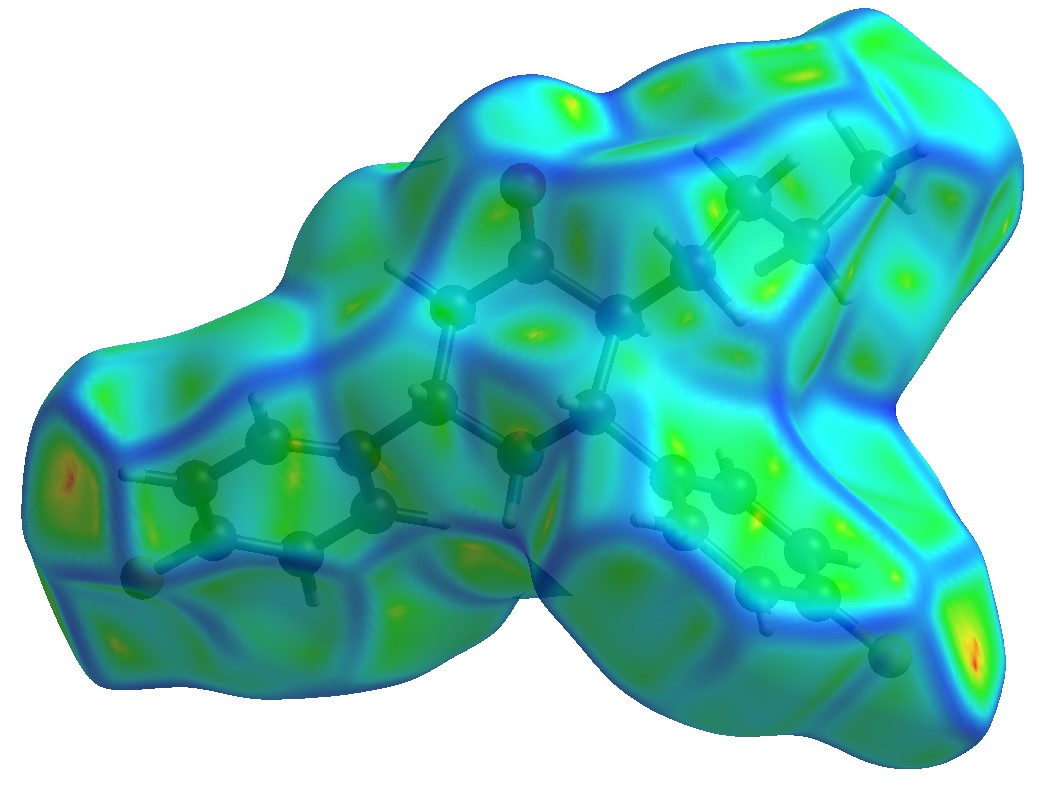
**

**Figure S2: Hirshfeld surface shape index for (I)**

**
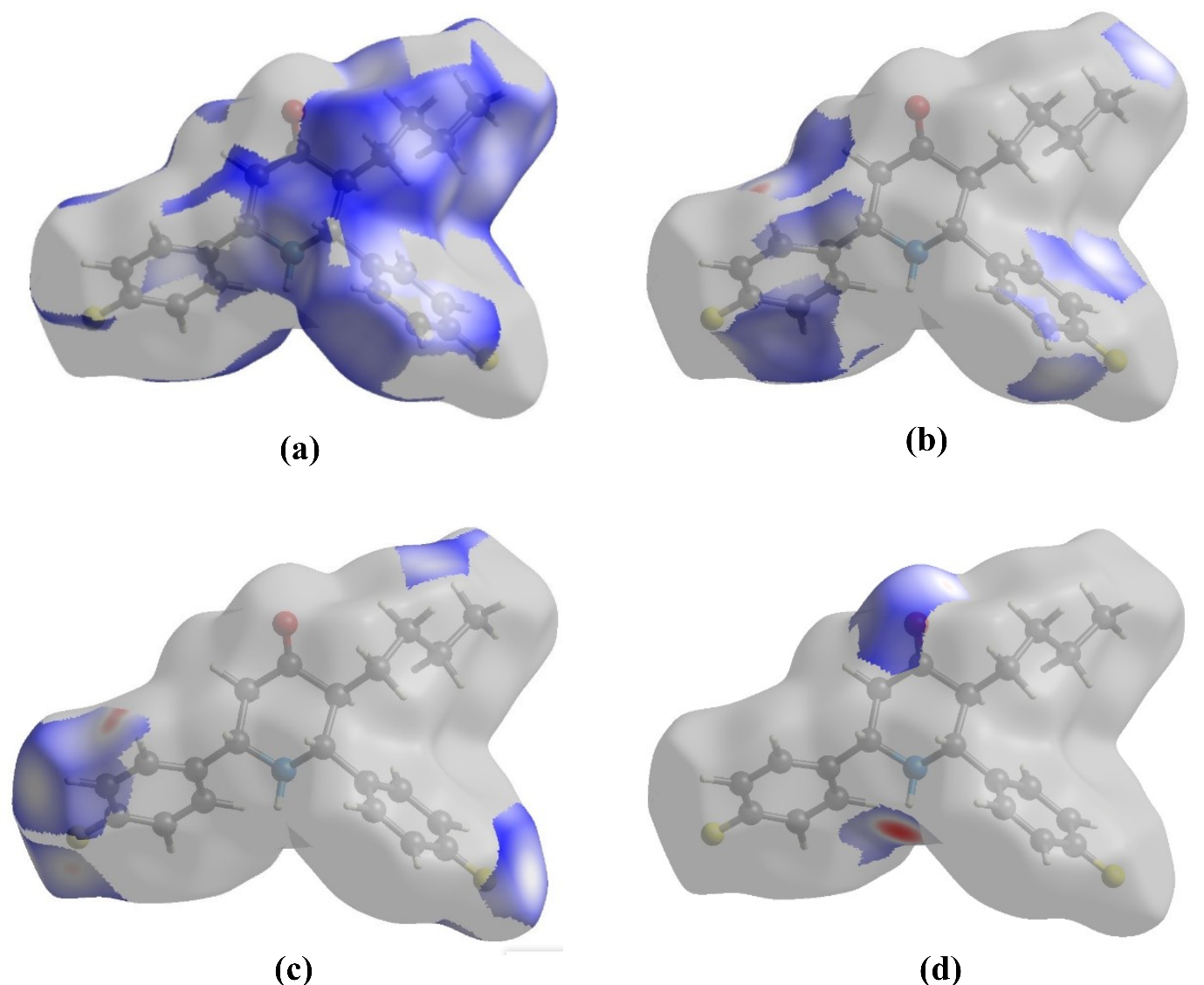
**

**Figure S3: Hirshfeld surface contact contributions for (I)**
